# Genetic Features of the Spontaneous Self-Compatible Mutant, ‘Jin Zhui’ (*Pyrus bretschneideri* Rehd.)

**DOI:** 10.1371/journal.pone.0076509

**Published:** 2013-10-07

**Authors:** Junkai Wu, Maofu Li, Tianzhong Li

**Affiliations:** 1 Laboratory of Fruit Tree Cell and Molecular Breeding, China Agricultural University, Beijing, China; 2 State Key Laboratory of Systematic and Evolutionary Botany, Institute of Botany, Chinese Academy of Sciences, Beijing, China; United States Department of Agriculture, United States of America

## Abstract

‘Jin Zhui’ is a spontaneous self-compatible mutant of ‘Ya Li’ (*Pyrus bretschneideri* Rehd. *S_21_S_34_*), the latter displaying a typical *S*-RNase-based gametophytic self-incompatibility (GSI). The pollen-part mutation (PPM) of ‘Jin Zhui’ might be due to a natural mutation in the pollen*-S* gene (*S_34_* haplotype). However, the molecular mechanisms behind these phenotypic changes are still unclear. In this study, we identified five *SLF* (*S-Locus F-box*) genes in ‘Ya Li’, while no nucleotide differences were found in the *SLF* genes of ‘Jin Zhui’. Further genetic analysis by *S-RNase* PCR-typing of selfed progeny of ‘Jin Zhui’ and ‘Ya Li’ × ‘Jin Zhui’ progeny showed three progeny classes (*S_21_S_21_*, *S_21_S_34_* and *S_34_S_34_*) as opposed to the two classes reported previously (*S_21_S_34_* and *S_34_S_34_*), indicating that the pollen gametes of ‘Jin Zhui’, bearing either the *S_21_*- or *S_34_*-haplotype, were able to overcome self-incompatibility (SI) barriers. Moreover, no evidence of pollen-*S* duplication was found. These findings support the hypothesis that loss of function of *S*-locus unlinked PPM expressed in pollen leads to SI breakdown in ‘Jin Zhui’, rather than natural mutation in the pollen*-S* gene (*S_34_* haplotype). Furthermore, abnormal meiosis was observed in a number of pollen mother cells (PMCs) in ‘Jin Zhui’, but not in ‘Ya Li’. These and other interesting findings are discussed.

## Introduction

Gametophytic self-incompatibility (GSI) is a genetic mechanism in many flowering plants that prevents self-fertilization and promotes cross-fertilization. In Solanaceae, Plantaginaceae and Rosaceae, GSI is controlled by a single multi-allelic *S* locus, which is comprised of the pistil-*S* and pollen-*S* genes [1]. The pistil-*S* gene encodes a polymorphic ribonuclease (S-RNase) essential for rejection of self-pollen, and operates through inhibiting the growth of pollen tubes that possess the same *S* allele [2]–[5]. The pollen-*S* locus itself is a cluster of pollen-expressed *S*-locus *F-box* genes named *SLF*/*SFBB* [6]–[14]. Recent evidence in *Petunia*, *Antirrhinum* and *Pyrus* support a model in which the SLF functions as a component of the SCF E3 ubiquitin ligase complex that interacts with non-self S-RNases, leading to their degradation through the ubiquitin 26S proteasome proteolytic pathway [15]–[17]. However, an alternative model based on studies of *Nicotiana* suggests the resistance to non-self S-RNase by its sequestration from vacuolar compartments in compatible pollen tubes [18]. Synthesizing the above two models, a comprehensive hypothesis involving both S-RNase degradation and compartmentalization was recently proposed [19], though many details are yet to be clarified.

In recent studies, the use of induced or spontaneous self-compatible mutants have supported *S-RNase* and *S*-locus *F-box* genes as the *S*-determinants in Rosaceae, although functional approaches based on transgenic experiments have been proven problematic in this family. For instance, Okada et al. [20] described a self-compatible Japanese pear cultivar, Osa-Nijisseiki (*S_2_S_4_*). The cultivar had a 236 kb region deleted which included the *S_4_-RNase* of *S4^sm^-*haplotype. Whereas Li et al. [21] found that self-compatibility of ‘Yan Zhuang’ (*Pyrus bretschneideri* Rehd.) was caused by a single amino acid mutation in *S_21_*-RNase. The European pears (*Pyrus communis*) ‘Abugo’ and ‘Ceremeno’ were found to be self-compatible where *S_21_*°-RNase protein was absent in the style [22]. In sour cherry (*Prunus cerasus*), a *Mu*-like element insertion upstream of *S_6m_-RNase* reduced the level of expression of *S-RNase*, correlating with reduced accumulation of S-RNase in the pistil and reversal of the rejection mechanism [23]. These results all support S-RNase as pistil-*S* determinants in Rosaceae. Meanwhile, self-compatible pollen-part mutants with non-functional SFB proteins have been widely reported in *Prunus*. In self-compatible sweet cherry (*Prunus avium*), the *SFB^4^* 4 bp deletion caused a frame-shift resulting in defective SFB^4^ transcripts lacking two hypervariable regions. Similarly, a self-compatible Japanese apricot (*Prunus mume*) mutant was likely caused by insertion in the *SFB^f^* coding region, leading to a defective SFB^f^ transcript that lacked the HVa and HVb containing C-terminus [9], [24]. These reports support the role of SFB as pollen-*S* determinants in *prunus*. Noteworthily, self-compatible pollen-part mutants with non-functional SLF/SFBB are little known in Solanaceae and the tribe Pyreae (apple, pear). Pollen-part self-compatibility (SC) in these species is due to the *S*-heteroallelic pollen effect [25]–[29]. The phenomenon is called competitive interaction (CI), since a pollen grain carrying two different pollen *S* alleles induces breakdown of pollen SI function [11], [27]. Recent functional analyses of *SLFs/SFBB*s in Solanaceae and *Pyrus* (Rosaceae) support a ‘non-self recognition by multiple factor SI system’ [14], [30]. Therefore, SC accessions reported in *Pyrus* are mostly related to *S*-allele duplications.

However, mutations in *S*-locus unlinked factors (also called modifier genes) that are required for GSI had also been associated with SC in Solanaceae and some genera of Rosaceae. In apricot cultivar ‘Canino’ (*S_2_S_C_*), a mutation was found in a modifier gene unlinked to the *S*-locus which independently caused the loss of pollen-*S* function [31], [32]. Results of Wu et al. [33] indicated that the SI breakdown in PPM ‘Katy’ apricot was associated with factors unlinked to the *S*-locus. Zuriaga et al. [34] reported another SC North-American apricot cv. Zaigers ‘Katy’, in which the mutated *S*-locus unlinked PPM was located at the distal end of chr.3. Pollen modifier factors have also been identified in *Petunia*, *Pyrus* and *Prunus*, such as the pollen-expressed Skp1-like1 proteins. As a modifier, Skp1-like1 proteins were proposed to be involved in a SCF complex [17], [35], [36]. Nevertheless, in *Pyrus*, the loss of function of *S*-locus unlinked PPM that leads to self-compatibility has never been reported. Therefore, the identification of *S*-locus unlinked PPM in *Pyrus* will be necessary to give a more complete picture of the self-incompatible mechanism.

‘Jin Zhui’ is a spontaneous self-compatible mutant of SI pear cultivar ‘Ya Li’. Li et al. [21] used genetic analysis of a small population to show that breakdown of SI in ‘Jin Zhui’ might be caused by pollen-*S_34_* mutation [21]. However, it is not clear what form of mutation led to the breakdown of SI in ‘Jin Zhui’. In this work, genetic populations of selfed ‘Jin Zhui’ progeny and crossed progeny with ‘Jin Zhui’ were constructed. We analyzed the self-compatible ‘Jin Zhui’ using genetic and molecular approaches, with the compiled evidence suggesting that loss of function of the *S*-locus unlinked PPM was probably involved in pollen-*S* function breakdown. Further mapping will be necessary to identify the underlying mutant *S*-locus unlinked PPM.

## Materials and Methods

### Ethics Statement

No specific permits were required for the described observation or field studies. For locations and activities partaken, a. no specific permissions were required; b. locations were not privately-owned or protected in any way and c. no endangered or protected species were involved.

### Plant Material

Five cultivars of Chinese pear (*Pyrus bretschneideri* Rehd.), ‘Ya Li’ (SI, *S_21_S_34_*), ‘Jin Zhui’ (SC, bud mutant of ‘Ya Li’ ), ‘Jin Feng’ (SI, *S_19_S_34_*), ‘Han Hong’ (SI, *S_27_S_34_*), ‘Han Xiang’ (SI, *S_12_S_31_*), ‘Jin Zhui’ selfed progeny, and crossed progeny derived from ‘Ya Li’ × ‘Jin Zhui’, ‘Ya Li’ × ‘Jin Feng’, ‘Jin Zhui’ × ‘Jin Feng’, ‘Jin Zhui’ × ‘Han Hong’, ‘Ya Li’ × ‘Han Hong’, ‘Han Xiang’ × ‘Jin Zhui’ were used in this study. The pistils and pollen were collected and stored at −80°C for DNA and RNA extraction. Flower buds from ‘Jin Zhui’ and ‘Ya Li’ were collected in spring at the stage ideal for meiotic studies, and root-tips for the observation of chromosomal numbers of ‘Jin Zhui’ selfed progeny were obtained from the germinated seeds.

The trees were planted on the farm of Dasungezhuang Orchard (Shunyi, Beijing, China), and seeds from self-pollination and cross-pollination were used for segregation analysis of *S-RNases*.

### Isolation of DNA and RNA

Genomic DNA from leaves and seedlings was isolated by the method described previously [37], [38], and then incubated with RNase I (Invitrogen, CA, USA) at 37°C for 2 hours to remove the RNA, after which it was quantified by spectrophotometer. RNA was extracted from leaves, styles, and pollen samples according to a modified SDS method [39] and digested with DNase I (TaKaRa, DaLian, China). RNA was detected by electrophoresis. Total RNA was used to synthesize first-strand cDNA using the SuperScript reverse transcriptase (Invitrogen, CA, USA) with Oligo-dT primer. The cDNA was then used as templates for PCR amplification.

### Genetic Linkage Analysis between Pollen-expressed *SLF* Alleles and *S-RNases*

Primers were screened from the PpSFBB primers reported by KaKui et al. [14]. Four primer pairs (Table 1) were chosen for amplifying the pollen-expressed-*SLF* alleles in pollen cDNAs from ‘Ya Li’ and ‘Jin Zhui’. PCR conditions were as follows: 20 µL reaction system, 50 ng genomic DNA, 2 µL 10 × Ex Taq buffer (including 2 mM MgCl_2_), 0.2 mM dNTPs, 10 pM of each primer and 0.2 unit Ex Taq DNA polymerase (TaKaRa). PCR products were examined by 1% agarose gel, and purified using the Gel Extraction Kit (BioDev-Tech, Beijing, China) and then cloned into the pMD18-T vector (TaKaRa). Four independent clones of each PCR product were chosen for DNA sequencing. Sequence alignment was performed with MEGA version 5.0 [40]. Phylogenetic analyses of the amino acid sequences of *SLF/SFBB* were carried out using the neighbor-joining method implemented in MEGA, with 1000 bootstrap replicates. Homologs were identified by BLASTN searches of the National Center for Biotechnology Information database (NCBI; [41]). Specific primer pairs for *SLF* alleles were designed for genetic linkage analysis of *SLF* alleles (Table 1). A segregating population of 30 individuals from ‘Ya Li’ (*S_21_S_34_*) × ‘Jin Feng’ (*S_19_S_34_*) was used.

**Table 1:** Sequences of oligonucleotide primers used in this study.

primer	Sequence(from5’ to 3′)	note
S_12_-F	AGTTGGTAATTATTTGGCCGAACG	This work
S_12_-R	TCAACCAATTCAGTCAATGATGTCC	This work
S_19_-F	GACCCAAAATATTGCAAGGCG	[17]
S_19_-R	TGGTTCTGTATTGGGGAAGACG	[17]
S_21_-F	ATATTGCAGGACAAGGAATCG	[17]
S_21_-R	ATATGGTGATCCGGGTAGAAAG	[17]
S_31_-F	AAGACCCAGAAGGTTGCAAGACACA	This work
S_31_-R	TTTCCAACTGGGGTTCGAGTATTTGC	This work
S_34_-F	ATGGGGATGACGGGGATGAT	[17]
S_34_-R	ATACTGAATACTATTGTTTGGGCAA	[17]
PActin-F	GGTGTCATGGTTGGTATGGGTC	[17]
PActin-R	TTCCGCAACCGCTTGAATAGA	[17]
PpSFBB1-F	GGACTTGTAGTTTGATTTAGTCTGG	[14]
PpSFBB1-R	AACGCCAGGCTATGAGTACTACTTC	[14]
PpSFBB2-F	TGGTGGTGTTTCCTATGTACAT	[14]
PpSFBB2-R	CATACAAATTAAATAGAAGAAAATG	[14]
PpSFBB3-F	AACCTTCATTATGATGTTAAGCC	[14]
PpSFBB3-R	TWAYGACAACAATAAGAAGTGATA	[14]
PpSFBB4-F	AAGAATTCTTGTGGAAAAAAAACT	[14]
PpSFBB4-R	CCTTATATCATGCATACAAATTAA	[14]
PbSLF3-S34-F	F: TTATCTTTCCATTTATAGTGACTAG	[17]
PbSLF3-S34-R	R: ACTATGATAAAATGTTCG	[17]
PbSLF6-S21-F	CATGAAAGTGAAACTCCTC	[17]
PbSLF6-S21-R	TACTTGAGATTTTCGGTACC	[17]
PbSLF1-S21-F	ATGTCCCTGGTGTATGAAAGT	This work
PbSLF1-S21-R	TATGTATTTCTCGCCATCGG	This work
PbSLF1-S34-F	ATGTCCCAGGTGCTTGAAAGG	This work
PbSLF1-S34-R	CAATCCCATTGCAATAGCCC	This work
PbSLF2-S34-F	AAATTTTGTCCAGGTTGCCAC	This work
PbSLF2-S34-R	GCAATCCAATAACAAAATCCCTTG	This work
PbSLF3-S34-F	TCTTCTATGCAATCCTTCG	This work
PbSLF3-S34-R	CAATAACAAAATCCCTTCG	This work

### Pollination Tests

Six cross-pollinations, ‘Ya Li’ × ‘Han Hong’, ‘Jin Zhui’ × ‘Han Hong’, ‘Han Xiang’ × ‘Jin Zhui’, ‘Ya Li’ × ‘Jin Zhui’, ‘Jin Zhui’ × ‘Jin Feng’, ‘Ya Li’ × ‘Jin Feng’ and two self-pollinations from ‘Ya Li’ and ‘Jin Zhui’, were performed in the field. Pollination was conducted as follows: all flowers were removed before anthesis except three per inflorescence, with the anthers in these three removed and insect-proof bags used to prevent accidental contamination by foreign pollen. Fruit set was recorded about 1 month later and fruits were collected up until harvest time, with the number of seeds and weight per fruit recorded.

### Pollen Viability and Germination Tests

Pollen viability was estimated as the percentage of pollen grains stained with 0.5% fluorescein diacetate (FDA). Fresh pollen grains were germinated on a solid medium (0.01% boric acid, 1% agar, 8% sucrose) in petri dish for 40 min at 28°C, and then the solid medium with germinated pollen grains was cut into small patches and placed on a glass slide. The germinated pollen grains were observed directly under a microscope (Nikon Eclipse 80i, Japan) for germination tests. For tests of pollen viability, FDA (2.5 mg/ml) was added to solid medium for 10 min, and then the pollen grains were observed under a microscope (Nikon Eclipse 80i, Japan). A total of 500 pollen grains were observed.

### Chromosome Preparation and Observation

Upon reaching the optimal stage for meiotic studies, flower buds of ‘Jin Zhui’ and ‘Ya Li’ were fixed in carnoy's fluid (3 parts of ethanol plus 1 part glacial acetic acid) for 24 hours and then stored in 70% ethanol at 4°C until used. Pollen mother cells were stained with 4′, 6-diamino-2-phenylindole (DAPI) before observation. The chromosome numbers of ‘Jin Zhui’ selfed progeny were observed from root-tip cells with at least 30 cells observed per sample, fresh root-tips were harvested when 1∼2 cm long from germinated seeds and pretreated in 0.002 M 8-Hydroxyquinoline for 2 hours at room temperature, and then fixed with cannoy solution for 24 hours. After washing twice with distilled water, the root-tips were cut and kept in a solution of 2% cellulose and 1% pectinase at 37°C for 2 hours. Materials were washed twice with distilled water before being fixed again with cannoy solution and then stained on a pre-cooled slide with 4′, 6-diamino-2-phenylindole pyrrolindone (DAPI). All chromosomal images were captured under the Olympus BX53 fluorescence microscope used with a microCCD camera.

## Results

### Molecular Identification of *SLFs* in ‘Ya Li’ and ‘Jin Zhui’

**Figure 1:**
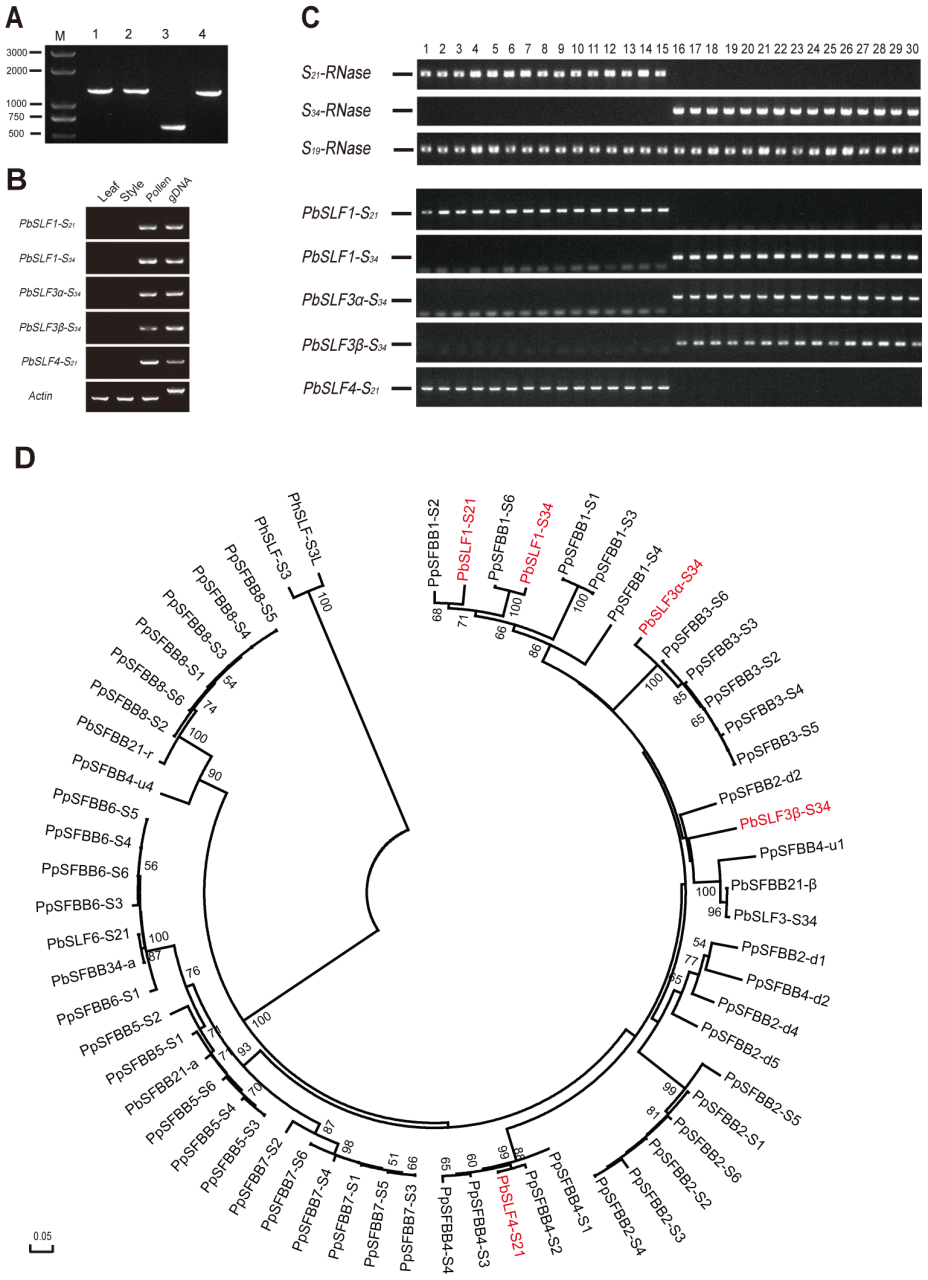
Molecular identification of *SLFs* in ‘Ya Li’. A. PCR products from genomic DNA of ‘Ya Li’ for *SLF* allele identification. Lanes 1–5 represent PCR products using primers PpSFBB1, PpSFBB2, PpSFBB3, and PpSFBB4, respectively. M, DNA marker. B. Expression of *PbSLFs* in leaf, style and pollen in ‘Ya Li’. RT-PCR was performed with *SLF*-specific primers and synthesized cDNA was used as templates. Pear *actin* gene was used as internal control. C. Linkage analysis of *PbSLFs* and *PbS-RNases*. Genomic DNAs from 30 crossed progeny of ‘Ya Li’ (*S_21_S_34_*) and ‘Jin Feng’ (*S_19_S_34_*) were used as templates for linkage analysis. *PbS_21_-RNase*, *PbS_34_-RNase* and *PbS_19_-RNase* (top panel) and *PbSLFs* (lower panel) were detected by PCR using gene specific primers. Lanes 1–30 represent a progeny population of 30 individuals. D. Phylogenetic tree of deduced amino acid sequences of PbSLFs and other SLFs/SFBBs. A neighbor-joining tree was constructed from 49 SLF/SFBB proteins from Japanese pear and 11 PbSLF proteins from *Pyrus bretschneideri*. PhSLF-S3 and PhSLF-S3L from *Petunia hybrida* were used as an outgroup. Numbers on the branches showed bootstrap values above 50% from 1000 bootstrap replicates. Eight types of the SLF/SFBB proteins were classified by Kakui et al. [14]. PbSLFs obtained in this study were marked in red.

PCR amplification of the *SLF* alleles was performed using four different primer pairs as used for *SFBB* allele identification by Kakui et al. [14] (Table 1). Single bands were obtained when amplifying *SLF* alleles from the genomic DNA of ‘Ya Li’ (*S_21_S_34_*) using each primer pair (Fig. 1 A). Direct sequencing of these PCR products validated the five *SLF* candidates. Specific primer pairs for each *SLF* were generated on the basis of sequence alignments of the cloned *SLFs* (Table 1). The PpSFBB4 primers were specific for the *S_21_*-haplotype of the *SLF* gene. The expression analysis of these *SLF* genes in different tissues showed they were all expressed specifically in pollen (Fig. 1 B).

To confirm whether the five *SLF* genes were linked to the *S* locus, a population of 30 progeny from ‘Ya Li’ × ‘Jin Feng’ was used. When 15 progeny carrying *S_19_S_21_* generated a single band which was absent in the other 15 progeny which carried *S_19_S_34_*, the *SLF* gene was assigned *S_21_*-haplotype-linkage. Similarly, if a single band only appeared in the progeny carrying *S_19_S_34_*, the *SLF* gene was assigned *S_34_*-haplotype-linked. Two of the *SLF* genes were detected in the *S_21_*-containing progeny only, and the other three were detected in the *S_34_*-containing progeny, suggesting linkage between the *PbSLFs* and *PbS-RNases* (Fig. 1 C). These *SLFs* were subject to phylogenetic analysis with *SLF*/*SFBBs* from Japanese pear and Chinese pear, based on which they were classified into three types: type-1, type-3 and type-4 according to Kakui et al. [14] (Fig. 1 D). Thus, they were named as *PbSLF1-S_21_* (GenBank accession number KC569798), *PbSLF1-S_34_* (GenBank accession number KC569799), *PbSLF3α-S_34_* (GenBank accession number KC569800), *PbSLF3β-S_34_* (GenBank accession number KC569801) and *PbSLF4-S_21_* (GenBank accession number KC569802).

To determine whether mutations or indels existed in the *SLFs* from ‘Jin Zhui’, seven *SLFs* including the five obtained in this study and two identified by Xu et al. [17] were cloned from pollen cDNA of ‘Jin Zhui’ and ‘Ya Li’. Comparative analysis of the seven *SLFs* showed no difference between ‘Ya Li’ and ‘Jin Zhui’ (data not shown).

### Fruit Set and Genetic Analysis of Selfed and Crossed Progeny of ‘Jin Zhui’

**Figure 2:**
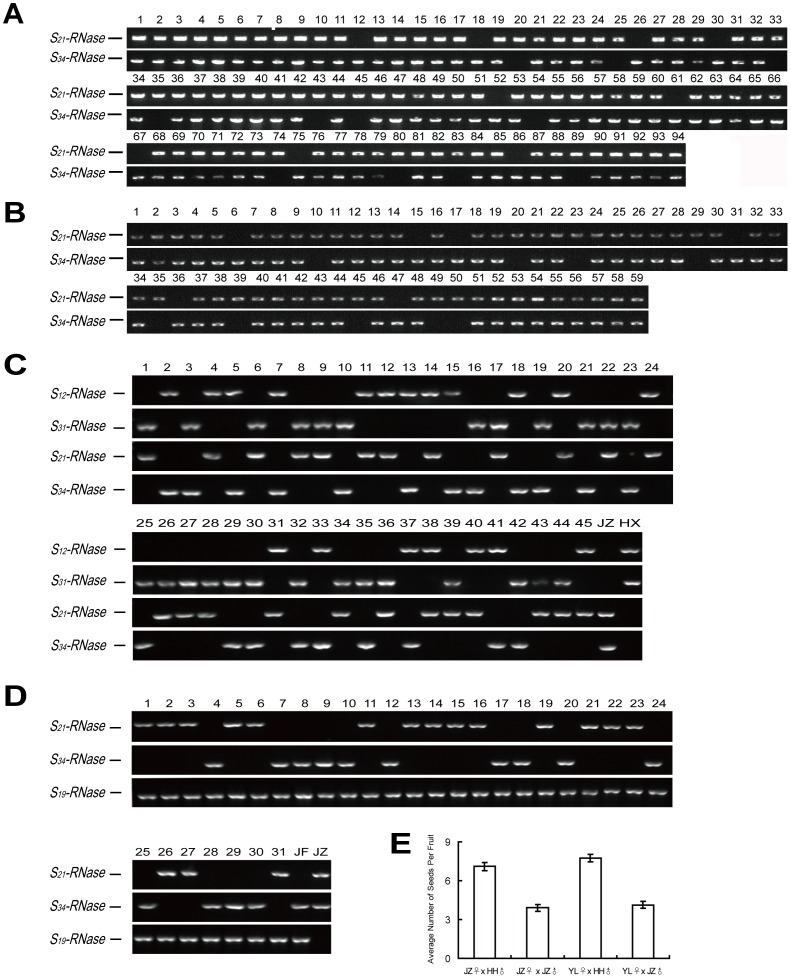
Segregation of *S-RNases* in selfed and crossed progeny of ‘Jin Zhui’. A. Segregation analysis of *S_21_*- and *S_34_-RNases* in selfed progeny of ‘Jin Zhui’. Lanes 1–94 represent a progeny population of 94 individuals. B. Segregation analysis of *S_21_-* and *S_34_-RNases* in crossed progeny between ‘Ya Li’ and ‘Jin Zhui’. Lanes 1–59 represents a progeny population of 59 individuals. C. Segregation analysis of *S*-alleles in crossed progeny between ‘Han Xiang’ and ‘Jin Zhui’. Lanes 1–45 represent a progeny population of 45 individuals. JZ, Jin Zhui; HX, Han Xiang. D. Segregation analysis of *S*-alleles in crossed progeny between ‘Jin Zhui’ and ‘Jin Feng’. Lanes 1–31 represent a progeny population of 31 individuals. JZ, Jin Zhui; JF, Jin Feng. E. Average numbers of seeds per fruit from selfed and crossed progeny of ‘Jin Zhui’. JZ, Jin Zhui; YL, Ya Li; HH, Han Hong.

Li et al. [21] found that ‘Jin Zhui’ was a pollen-part mutant of ‘Ya Li’. Genetic analysis of 29 selfed progeny of ‘Jin Zhui’ indicated the SI breakdown in ‘Jin Zhui’ might be due to the pollen-*S* of the *S_34_-*haplotype. To further analyze the nature of the self-compatible mutant, pollination tests were performed. ‘Ya Li’ and ‘Jin Zhui’ were self-pollinated and reciprocally pollinated. The results from pollination tests showed that ‘Jin Zhui’, which had functional style and nonfunctional pollen, was self-compatible, while ‘Ya Li’, which had functional style and pollen, was self-incompatible (Table 2). Further genetic analysis of larger populations of selfed and crossed progeny of ‘Jin Zhui’ was performed. app:addword:respectiveSegregation of *S-RNases* in selfed progeny of ‘Jin Zhui’ and crossed progeny between ‘Ya Li’ and ‘Jin Zhui’ was investigated by PCR using the *S*-haplotype-specific *S-RNase* primers. The results showed that both the selfed progeny of ‘Jin Zhui’ and the crossed progeny between ‘Ya Li’ and ‘Jin Zhui’ consisted of three *S*-genotypes: S*_21_*S*_21_*, *S_21_S_34_* and *S_34_S_34_* (Fig. 2 A, B and Table 3). The results indicated that pollen carrying both pollen *S*-alleles could reach the ovule and achieve self-fertilization, which suggested that the pollen-part mutation in ‘Jin Zhui’ was unlinked to the *S*-locus.

**Table 2:** Fruit setting rates in self- or cross-pollination of three pear cultivars.

Crosses	Pollinated flowers	Fruits set	Fruit-set per. (%)	Pollinated inflorescence No.
‘Ya Li’ selfed	117	3	2.6	39
‘Jin Zhui’ selfed	150	119	79.3	50
‘Jin Zhui’♀ × ‘Ya Li’♂	106	5	4.7	36
‘Ya Li’♀ × ‘Jin Zhui’♂	150	124	82.7	53
‘Ya Li’♀ × ‘Han Hong’♂	133	103	77.4	50
‘Jin Zhui’♀ × ‘Han Hong’♂	120	104	86.7	42

**Table 3:** Segregation of *S*-genotypes in progeny of different self- or cross-pollinations.

Crosses	Total	*S*-genotypes observed in progeny									Expectedsegregation ratio^a^	?^2^ *P*-value
		*S_21_S_21_ S_21_S_34_ S_34_S_34_ S_19_S_21_ S_19_S_34_ S_12_S_21_ S_12_S_34_ S_21_S_31_ S_31_S_34_*										
‘Jin Zhui’ selfed	94	11	74	9	–	–	–	–	–	–	1∶2∶1	31.11 (*P*<0.01)
‘Ya Li’ × ‘Jin Zhui’	59	9	44	6	–	–	–	–	–	–	1∶2∶1	14.56 (*P*<0.01)
‘Jin Zhui’ × ‘Jin Feng’	31	–	–	–	17	14	–	–	–	–	1∶1	0.29 (*P*>0.05)
‘Han Xiang’ × ‘Jin Zhui’	45	–	–	–	–	–	10	9	14	12	1∶1;1∶1	1.31 (*P*>0.05)

a Expected ratios for a single mutation unlinked to the *S*-locus.

To further validate these observations, additional crosses were performed and analyzed (Fig. 2 C, D and Table 3). The crossed progeny of ‘Han Xiang’ × ‘Jin Zhui’ and ‘Jin Zhui’ × ‘Jin Feng’ fell into four classes (*S_12_S_21_:S_12_S_34_*:*S_21_S_31_*:*S_31_S_34_*) and two classes (*S_19_S_34_: S_19_S_21_*) by *S-RNase* genotyping, respectively. The observed ratios for *S*-genotype segregations fit with the expected ratios (χ2 values of 1.31, *P*>0.05 and 0.29, *P*>0.05). These results supported self-compatibility (SC) in ‘Jin Zhui’ was associated with *S*-locus unlinked PPM rather than *S*-allele duplications.

The homozygotes from selfed progeny of ‘Jin Zhui’ and crossed progeny of ‘Ya Li’ × ‘Jin Zhui’ were much fewer in number than heterozygotes (Table 3). The average number of seeds per fruit produced from selfed progeny of ‘Jin Zhui’ and crossed progeny of ‘Ya Li’ × ‘Jin Zhui’ was 3.91 and 4.13, respectively. This was much lower than the two cross-pollinations ‘Jin Zhui’ × ‘Han Hong’ (7.09) and ‘Ya Li’ × ‘Han Hong’ (7.75) (Fig. 2 E). The segregation ratios of the three classes (*S_21_S_21_*:*S_21_S_34_*:*S_34_S_34_*) from selfed progeny of ‘Jin Zhui’ and crossed progeny between ‘Ya Li’ and ‘Jin Zhui’ were 9∶74∶11 and 6∶45∶9, respectively. These segregation ratios did not fit the expected ratios of 1∶2∶1, with χ2 values of 31.11, *P*<0.01 and 14.56, *P*<0.01 (Table 3). The segregation distortion showed that there were significantly fewer homozygotes in the progeny than heterozygotes, and the reduction in homozygosity was probably due to postzygotic selection.

### Significant Difference in Abortive Pollen between ‘Jin Zhui’ and ‘Ya Li’

**Figure 3:**
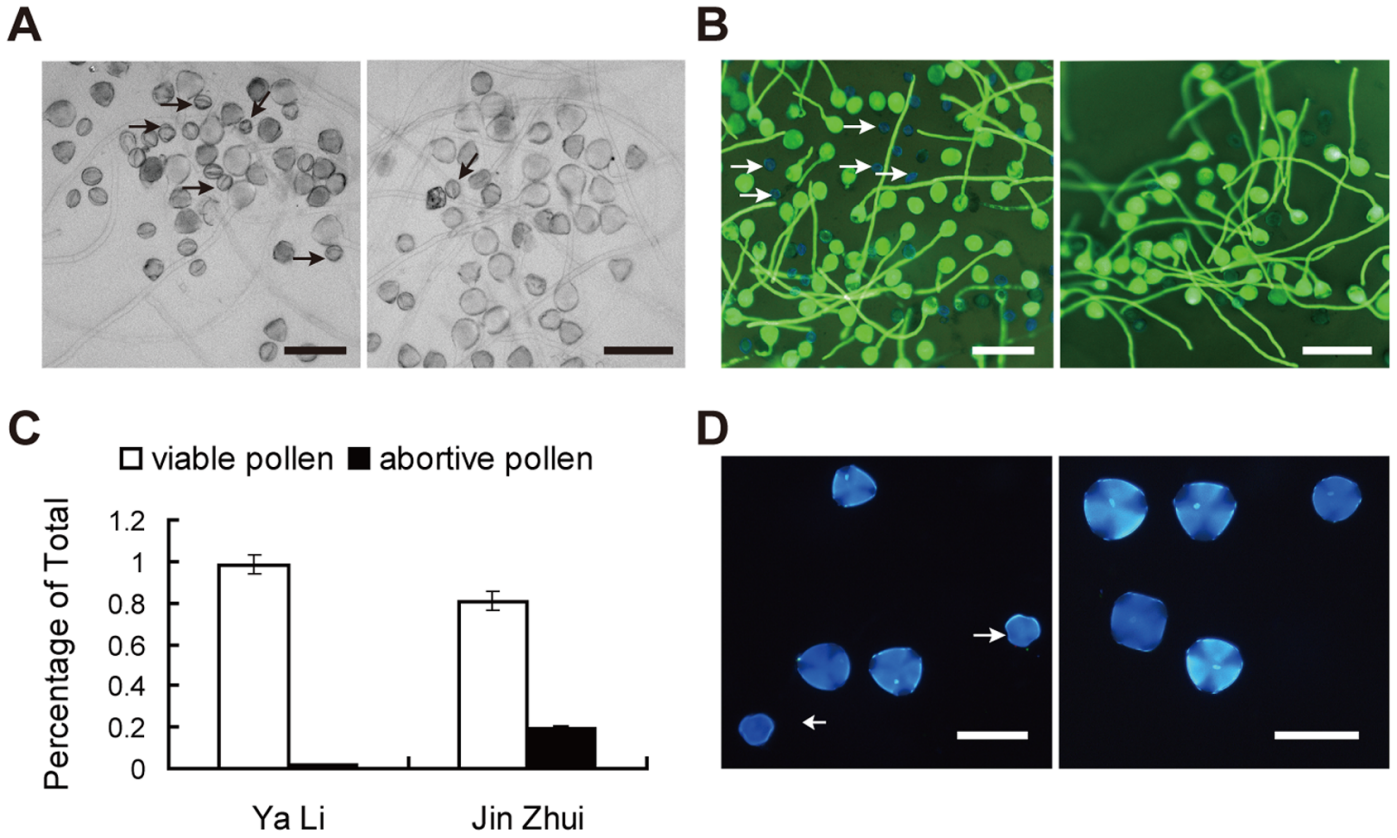
Significant difference in abortive pollen grains between ‘Jin Zhui’ and ‘Ya Li’. A. Pollen grains germination of ‘Jin Zhui’(left) and ‘Ya Li’ (right). Abortive pollen grains without hydration and germination are indicated with arrows. Scale bar: 100 µm. B. FDA staining of germinated pollen grains from ‘Jin Zhui’ (left) and ‘Ya Li’ (right). Abortive pollen grains not stained by FDA are indicated by arrows. Scale bar: 100 µm. C. Percentages of abortive pollen grains in FDA staining between ‘Jin Zhui’ and ‘Ya Li’. A total of 500 fresh pollen grains were examined in each case D. DAPI staining of pollen grains from ‘Jin Zhui’ (left) and ‘Ya Li’ (right). Abortive pollen grains without apparent nucleus are indicated by arrows. Scale bar: 50 µm.

To investigate the pollen-part mutation of ‘Jin Zhui’, we observed the pollen germination ability of ‘Jin Zhui’ and ‘Ya Li’ (Fig. 3 A), where a significant difference was found. The number of abortive pollen grains was found to be much increased in ‘Jin Zhui’, with the abortive pollen grains incapable of both hydration and germination. The abortive pollen grains of ‘Jin Zhui’ and ‘Ya Li’ could not be stained by FDA, indicating they were inviable (Fig. 3 B). The percentage of abortive pollen grains in ‘Jin Zhui’ (about 20%) was much higher than in ‘Ya Li’ (about 1.6%) (Fig. 3 C), which was consistent with the results observed during pollen germinations. Further DAPI staining showed that the abortive pollen grains had no apparent nucleus (Fig. 3 D), and that they had already degenerated into shells. The increase in these types of abortive pollen grains in ‘Jin Zhui’ implied partial disruption of normal meiosis during the development of pollen.

### Abnormal Meiosis of Pollen Mother Cells (PMCs) in ‘Jin Zhui’

**Figure 4:**
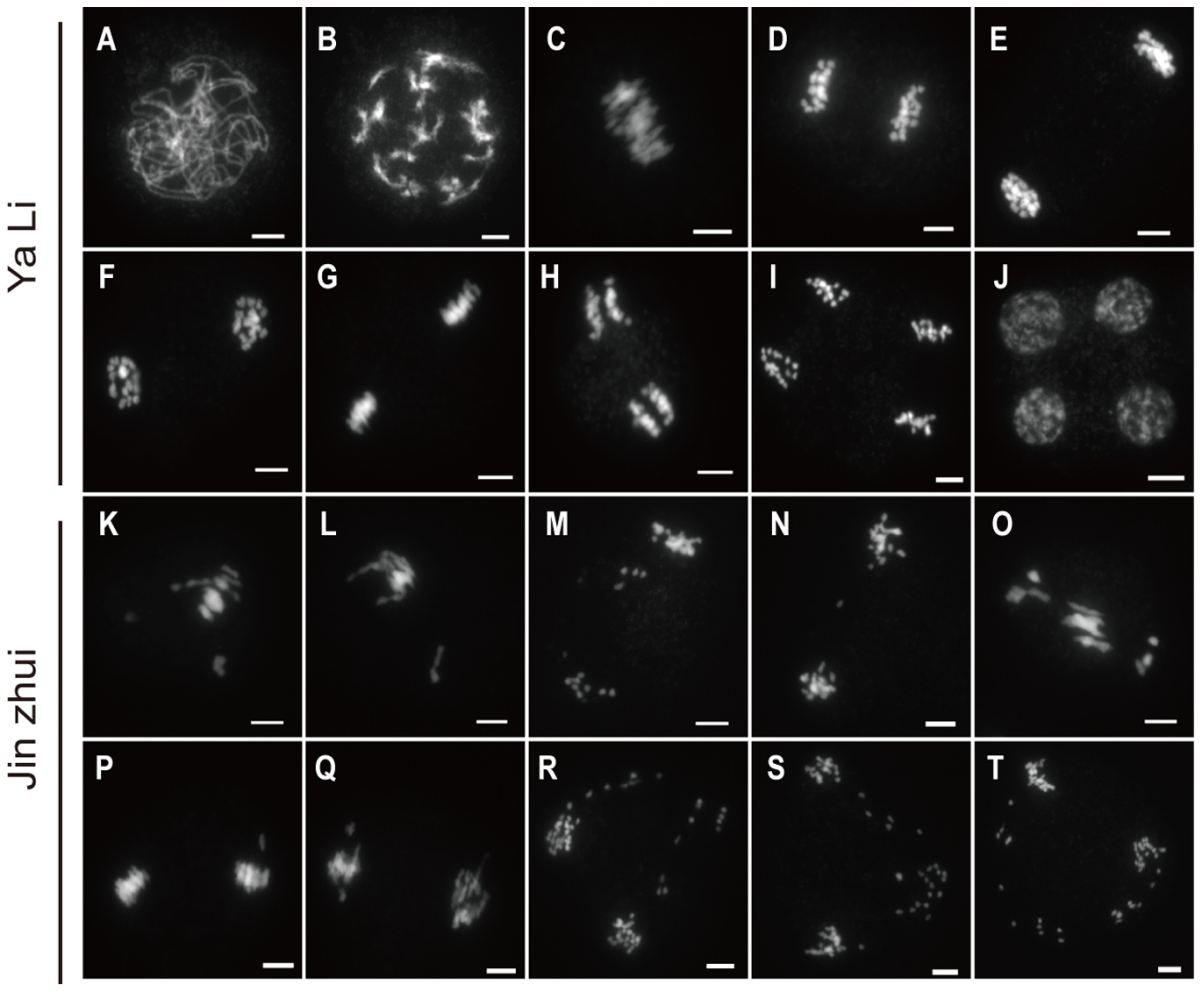
Meiosis of ‘Ya Li’ and ‘Jin Zhui’. (A–J). DAPI staining of ‘Ya Li’ PMCs during meiosis. (A)–(J) show pachytene, diakinesis, metaphase I, anaphase I, telophase I, prophase II, metaphase II, anaphase II, telophase II and tetrad, respectively. (K–T) DAPI staining of ‘Jin Zhui’ PMCs during meiosis. (K) and (L) show univalents in metaphase I, (M), (N) and (O) laggard chromosomes at anaphase I, (P) and (Q) univalents in metaphase II, (R), (S) and (T) laggard chromosome and unbalanced separation in anaphase II, respectively. Scale bar: 5 µm.

**Figure 5:**
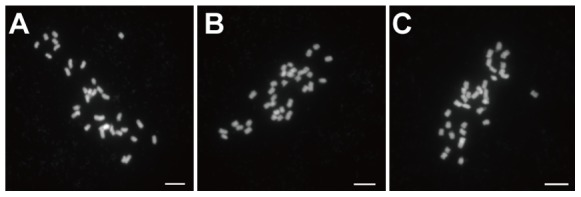
Normal chromosome numbers in selfed progeny of ‘Jin Zhui’. A, 34 chromosomes of an *S_21_S_21_* homozygote representing a total of 12 progeny examined. B. 34 chromosomes of an *S_21_S_34_* heterozygote representing a total of 21 progeny examined. C. 34 chromosomes of an *S_34_S_34_* homozygote representing a total of 13 progeny examined. Scale bar: 5 µm.

In order to investigate the abortive pollen grains in ‘Jin Zhui’, we analyzed the meiotic behavior of pollen mother cells (PMCs). As a self-incompatible cultivar, ‘Ya Li’ had normal meiotic behavior (Fig. 4 A–J). ‘Ya Li’ possesses 34 chromosomes, which during meiotic prophase I, undergo leptotene (not shown), zygotene (not shown), pachytene (Fig. 4 A), diplotene (not shown) and finally, the 17 bivalents condense through diakinesis (Fig. 4 B). The 17 bivalents then distribute on the equatorial plate at metaphase I (Fig. 4 C). During anaphase I, each group of homologous chromosomes separated from each other (Fig. 4 D), with each group reaching one pole of the cell (Fig. 4 E). Through meiotic prophase II (Fig. 4 F) and metaphase II (Fig. 4 G), the sister chromatids in each group were then separated in anaphase II (Fig. 4 H), generating four pools of 17 chromosomes (Fig. 4 I), which gave rise to four tetrads of microspores (Fig. 4 J). As a self-compatible pollen-part mutant of ‘Ya Li’, ‘Jin Zhui’ had abnormal meiosis in a number of PMCs (Fig. 4 K–T). Univalents appeared at metaphase I (Fig. 4 K and L), and laggards prevailed during anaphase I (Fig. 4 M, N, and O). During metaphase II, univalents also appeared (Fig. 4 P and Q), and then laggards and unbalanced separation were observed in anaphase II (Fig. 4 R, S and T). Unbalanced separation produced many abortive pollen grains which had no nucleus nor vitality. The number of meiotic abnormalities in ‘Jin Zhui’ PMCs was recorded (Table 4), and showed that abnormal meiosis existed in a number of PMCs of ‘Jin Zhui’. To test whether the abnormal meiosis affected the selfed progeny, we observed chromosome numbers of selfed progeny from ‘Jin Zhui’. Both the homozygotes and heterozygotes in the progeny had the usual (34) number of chromosomes (Fig. 5). Though abnormal meiosis was found in ‘Jin Zhui’ PMCs, the selfed progeny from ‘Jin Zhui’ had normal chromosome numbers.

**Table 4:** Meiotic abnormalities in ‘Jinzhui’.

Phases	Total number of cells	Number of abnormal cells	Abnormalities	Percentage of abnormal cells (%)
Metaphase I	625	283	univalents	45.28
Anaphase I	731	172	Laggards	23.53
Metaphase II	563	169	univalents	30.01
Anaphase II	612	244	laggards and unbalanced separation	39.87

## Discussion

### Identification and Comparative Analysis of *SLF* Genes between ‘Ya Li’ and ‘Jin Zhui’

In gametophytic self-incompatible (GSI) species of Solanaceae, Rosaceae and Plantaginaceae, multiple *F-box* genes within the *S*-locus region have been reported [6]–[8], [12], [42]. Through mutant analysis and transgenic approaches, the pollen *S* genes had been determined experimentally [9]–[11], [24], [43]. In *Petunia* and *Pyrus*, multiple SLFs/SFBBs as pollen *S* factors were proven to collaborate in recognizing non-self *S*-RNases and mediate their degradation [14], [30]. In previous studies, mutations or indels in pollen-*S* genes were always considered a reasonable explanation for self-compatible PPM, where Li et al. [21] reported that ‘Jin Zhui’ was a pollen-part mutant. The breakdown of self-incompatibility (SI) in ‘Jin Zhui’ might be caused by mutant pollen-*S_34_* genes. Our study on pollen-part mutation of ‘Jin Zhui’ should help us to understand the function of the pollen-*S* gene. We cloned five *F-box* genes from pollen cDNA of ‘Ya Li’, with linkage analysis showing them to be *S-Locus F-box* (*SLF*) genes. However, comparative analysis of seven *SLFs,* including the two *SLFs* identified by Xu et al. [17], showed no differences between ‘Ya Li’ and ‘Jin Zhui’. Therefore, these seven *SLFs* were probably not directly responsible for the SI breakdown of ‘Jin Zhui’. Subsequent genetic analysis further supported this hypothesis.

### Self-compatibility in ‘Jin Zhui’ is Associated with *S*-locus Unlinked PPM

‘Ya Li’ (*S_21_S_34_*) is a traditional Chinese pear (*Pyrus bretschneideri*) cultivar exhibiting self-incompatibility, and ‘Jin Zhui’ (*S_21_S_34_*) is a pollen-part mutant of ‘Ya Li’. Genetic analysis of 29 selfed progeny in ‘Jin Zhui’ showed that the pollen *S_34_* allele might be mutated in ‘Jin Zhui’ [21].

To investigate the genetics of SC in ‘Jin Zhui’, genetic populations were constructed. ‘Jin Zhui’ (*S_21_S_34_*) was self-pollinated and reciprocally crossed with ‘Ya Li’ (*S_21_S_34_*). It is noteworthy that ‘Jin Zhui’ pollen tubes bearing either the *S_21_*- or the *S_34_*-haplotype were able to grow through ‘Jin Zhui’ and ‘Ya Li’ pistils and complete fertilization, producing three *S*-genotype classes (*S_21_S_21_*, *S_21_S_34_* and *S_34_S_34_*) instead of the two classes observed previously (*S_21_S_21_* and *S_21_S_34_*). However, no progeny were obtained in the reciprocal cross when using ‘Jin Zhui’ as the female parent. These results should support the PPM in ‘Jin Zhui’ was unlinked to the *S*-locus. The crosses between ‘Han Xiang’ (*S_12_S_31_*) and ‘Jin Zhui’ (*S_21_S_34_*), ‘Jin Zhui’ (*S_21_S_34_*) and ‘Jin Feng’ (*S_19_S_34_*) reinforced this conclusion.

Interestingly, in the ‘Jin Zhui’ × ‘Jin Zhui’ and ‘Jin Zhui’ × ‘Ya Li’ populations the number of seedlings homozygous for both the *S_21_*- and the *S_34_*-haplotype is significantly lower than that for the heterozygote *S_21_S_34_* (see Table 3). Some explanations for this phenomenon may include linkage in coupling between the mutated allele of the *S*-locus unlinked PPM and the *S*-allele, or postzygotic selection against homozygous embryos. In the case of the former, the segregation ratios observed in different populations do not support linkage between the mutated factor and the *S*-locus. Whereas postzygotic selection would explain the significantly reduced numbers of *S_21_S_21_* and *S_34_S_34_* genotypes, and could also be a reasonable explanation that no *S_21_S_21_* homozygotes were detected previously by Li et al. [21] in a small population from selfed progeny of ‘Jin Zhui’.

In Solanaceae, self-compatible pollen-part mutants may arise from *S*-allele duplications located in a centric fragment, in a non-*S* chromosome, or linked to the *S*-locus leading to the formation of *S*-heteroallelic pollen [27]. According to the segregations obtained in the crosses (including ‘Jin Zhui’ selfed, ‘Ya Li’ × ‘Jin Zhui’, ‘Jin Zhui’ × ‘Jin Feng’, ‘Han Xiang’ × ‘Jin Zhui’) performed here, *S*-allele duplications did not seem likely in ‘Jin Zhui’ (all descendants should have had the *S_21_S_34_* genotype) (Fig. 2, Table 3). *S*-allele duplications may also result from polyploidy, but ‘Jin Zhui’ was confirmed as diploid by chromosome observation in selfed progeny (Fig. 5). These results rule out competitive interaction resulting from *S*-heteroallelic pollen as the cause of SC in ‘Jin Zhui’. Taken together, these results supported the hypothesis that an *S*-locus unlinked PPM was required in GSI. Loss of function in this *S*-locus unlinked PPM was responsible for the SI breakdown of ‘Jin Zhui’.

### Possible Role of the *S*-locus Unlinked PPM in Meiosis

Abnormal meiotic behaviors in PPM ‘Jin Zhui’ were observed in many PMCs (Fig. 4 and Table 4). Univalents, laggards and unbalanced separation were detected during the process of meiosis (Fig. 4). In anaphase II, unbalanced separation produced a number of abnormal microspores, which were degraded into shells (Fig. 3). Meanwhile, to test whether the abnormal meiosis affected the selfed progeny, we observed chromosome numbers of selfed progeny from ‘Jin Zhui’, and found that both the homozygotes and heterozygotes in the progeny had the usual 34 chromosomes (Fig. 5). Though abnormal meiosis was found in ‘Jin Zhui’ PMCs, the selfed progeny from ‘Jin Zhui’ had normal chromosome numbers and were not influenced by abnormal meiosis. Abnormal meiosis in PMCs was consistently caused by related mutant genes. In an *Arabidopsis* male sterile mutant, abnormal meiosis caused by a Ds insertion in the *SKP1-LIKE1* gene was reported [44]. While the SKP1-LIKE1 homolog SSK (SLF-interacting Skp1-like1) was identified as a putative canonical SCF^SLF^ complex, and proposed to degrade non-self S-RNase in the degradation model of *Pyrus, prunus* and *Antirrhinum* [17], [35], [36], [45]. In this work, we suspect the mutant *S*-locus unlinked PPM might influence the pollen development of ‘Jin Zhui’. Two *SSKs*, which had been identified in ‘Ya Li’ [17], were cloned from pollen cDNAs of ‘Jin Zhui’ and ‘Ya Li’, but no nucleotide difference was found (data not shown). Further studies focusing on the identification of *S*-locus unlinked PPM should help us to gain deeper insights into self-incompatibility in *Pyrus*.
